# Allergic Responses Induced by the Immunomodulatory Effects of Nanomaterials upon Skin Exposure

**DOI:** 10.3389/fimmu.2017.00169

**Published:** 2017-02-16

**Authors:** Yasuo Yoshioka, Etsushi Kuroda, Toshiro Hirai, Yasuo Tsutsumi, Ken J. Ishii

**Affiliations:** ^1^Vaccine Creation Project, BIKEN Innovative Vaccine Research Alliance Laboratories, Research Institute for Microbial Diseases, Osaka University, Suita, Osaka, Japan; ^2^BIKEN Center for Innovative Vaccine Research and Development, The Research Foundation for Microbial Diseases of Osaka University, Suita, Osaka, Japan; ^3^Laboratory of Nano-Design for Innovative Drug Development, Graduate School of Pharmaceutical Sciences, Osaka University, Suita, Osaka, Japan; ^4^The Center for Advanced Medical Engineering and Informatics, Osaka University, Suita, Osaka, Japan; ^5^Laboratory of Vaccine Science, Immunology Frontier Research Center, World Premier International Research Center, Osaka University, Suita, Osaka, Japan; ^6^Department of Dermatology and Immunology, University of Pittsburgh, Pittsburgh, PA, USA; ^7^Laboratory of Toxicology and Safety Science, Graduate School of Pharmaceutical Sciences, Osaka University, Suita, Osaka, Japan; ^8^Laboratory of Adjuvant Innovation, National Institutes of Biomedical Innovation, Health and Nutrition, Ibaraki, Osaka, Japan

**Keywords:** adjuvant, allergy, aluminum salts, biodistribution, metal allergy, nanomaterial, sensitization, skin

## Abstract

Over the past decade, a vast array of nanomaterials has been created through the development of nanotechnology. With the increasing application of these nanomaterials in various fields, such as foods, cosmetics, and medicines, there has been concern about their safety, that is, nanotoxicity. Therefore, there is an urgent need to collect information about the biological effects of nanomaterials so that we can exploit their potential benefits and design safer nanomaterials, while avoiding nanotoxicity as a result of inhalation or skin exposure. In particular, the immunomodulating effect of nanomaterials is one of most interesting aspects of nanotoxicity. However, the immunomodulating effects of nanomaterials through skin exposure have not been adequately discussed compared with the effects of inhalation exposure, because skin penetration by nanomaterials is thought to be extremely low under normal conditions. On the other hand, the immunomodulatory effects of nanomaterials *via* skin may cause severe problems for people with impaired skin barrier function, because some nanomaterials could penetrate the deep layers of their allergic or damaged skin. In addition, some studies, including ours, have shown that nanomaterials could exhibit significant immunomodulating effects even if they do not penetrate the skin. In this review, we summarize our current knowledge of the allergic responses induced by nanomaterials upon skin exposure. First, we discuss nanomaterial penetration of the intact or impaired skin barrier. Next, we describe the immunomodulating effects of nanomaterials, focusing on the sensitization potential of nanomaterials and the effects of co-exposure of nanomaterials with substances such as chemical sensitizers or allergens, on the onset of allergy, following skin exposure. Finally, we discuss the potential mechanisms underlying the immunomodulating effects of nanomaterials by describing the involvement of the protein corona in the interaction of nanomaterials with biological components and by presenting recent data about the adjuvant effects of well-characterized particle adjuvant, aluminum salt, as an example of immunomodulatory particulate.

## Introduction

Recently, advances in nanotechnology have made possible the design and production of many engineered nanomaterials—nanoparticles, nanofibers, and nanosheets—which are defined as materials with structures having at least one dimension less than 100 nm (1, 2). These products have become indispensable in various fields, such as electronics, foods, cosmetics, and medicines, because nanomaterials have unique physicochemical properties and exert innovative functions compared with conventional larger particles; these properties and functions include enhanced electrical conductivity, tensile strength, and chemical reactivity, and stem from an increase in the surface area per unit weight compared with a larger amount (>100 nm) of the same material (3, 4). However, with the increasing use of nanomaterials, concerns about their safety, termed nanotoxicity, have been raised, specifically that the innovative functions of nanomaterials, such as high chemical reactivity and high tissue penetration, due to their small size might make them hazardous in some situations (5, 6). For example, our group has shown that intravenous injection of a large amount of silica (SiO_2_) nanoparticles induced pregnancy complications in mice, although it should be noted that the level of exposure used in the study is not representative of real-world human exposure (7). The health risks of engineered nanomaterials to humans have also been considered (8–10). Among the nanotoxic effects, those on host immunity are of particular interest, because the immune cells recognize foreign substances as part of the body’s defenses, when those substances enter the body. Therefore, there have been many reports about the immunomodulating effects, both immune-activating and -suppressing effects, of nanomaterials *in vitro* and *in vivo* (11–13). To fully utilize the potential benefits of nanomaterials and design safer nanomaterials, it is essential for us to collect more information about nanotoxicity, because intentional and unintentional exposure to nanomaterials is unavoidable in our everyday life.

Our skin is exposed to nanomaterials in many situations, because nanomaterials are contained in cosmetics and other skincare products. For example, some nanoparticles, especially Zinc oxide (ZnO) and titanium dioxide (TiO_2_) nanoparticles, have been used in sunscreens since the 1980s, because they have better ultraviolet (UV) protective properties than larger particles (14). SiO_2_ nanoparticles are used in a wide variety of cosmetics as an anti-setting agent (15). We are also exposed to silver (Ag) nanoparticles through our everyday lives because Ag nanoparticles have been widely applied to consumer products such as clothing, antibacterial sprays, detergent, socks, and shoes for antimicrobial purposes (16). Therefore, an understanding of the absorption rate of nanomaterials after exposure *via* the skin has attracted increasing attention over the past few years, because it is important to consider the immunomodulatory effects of nanomaterials on the skin. In addition, because nanomaterials can interact with other substances easily (17), we must not forget that exposure to nanomaterials *via* skin often occurs simultaneously with exposure to other chemical compounds and allergens, such as foods and pollen and that this interaction might modulate the antigenicity of these compounds. Many recent reports have shown that skin is an important site for the onset of allergy (18, 19). For example, several reports have shown that transdermal exposure to food allergens can induce Th2-type immune responses and be sufficient to sensitize mice (20–22). Furthermore, individuals who used a facial soap containing hydrolyzed wheat protein were presumed to be sensitized to this protein (23, 24). Given these findings, there is an urgent need to understand the immunomodulatory effects of nanomaterials upon skin exposure, particularly effects that may lead to the onset or aggravation of allergy. However, while there have been many studies examining the nanotoxicity of nanomaterials to the respiratory system, there is a lack of knowledge about nanotoxicity following skin exposure to nanomaterials, especially the immunomodulating effects.

In this review, we summarize our current understanding of the skin penetration of nanomaterials and the immunomodulating effects of nanomaterials, focusing on the skin penetration of nanomaterials, the sensitization potential of nanomaterials, and the effects of co-exposure of nanomaterials with allergens on the onset of allergy upon skin exposure. In addition, we discuss potential mechanisms underlying the immunomodulating effects of nanomaterials by describing the involvement of the protein corona in the interaction of nanomaterials with complement proteins and by presenting recent study about the adjuvant effects of aluminum salts, which are well characterized in basic immunology.

## Skin Structure and Penetration of Skin by Nanomaterials

The skin is composed of several barriers that prevent foreign substances from penetrating the body (25, 26). Healthy skin is divided into the epidermis and the dermis. In addition, there are two physical barriers in the epidermis: the stratum corneum, the outmost layer of the epidermis, and tight junctions, which are intercellular junctions that seal adjacent keratinocytes in the stratum granulosum below the stratum corneum (25, 26). It is generally believed that molecules, other than small lipophilic molecules (<500 Da), are unable to penetrate healthy skin due to these barrier functions (27). The skin also contains hair follicles and sebaceous glands (28). Hair follicles extend into the dermis and might provide a means for penetration and absorption of compounds into the skin. Therefore, hair follicles may play an important role as a potential reservoir and penetration route for topically applied substances. It is also suggested that hair follicles have important functions in immune responses such as those regulating the trafficking of antigen presenting cells (29). In addition, many immune cells such as antigen presenting cells [e.g., Langerhans cells (LCs) in the epidermis and dermal dendritic cells in the dermis] and leukocytes are present in the skin to protect the body from external substances (30). Recently, tape stripping of murine skin showed that activated LCs could elongate their dendrites above the tight junctions of keratinocytes and take up antigens on the surface of the skin (31, 32).

**Figure 1:**
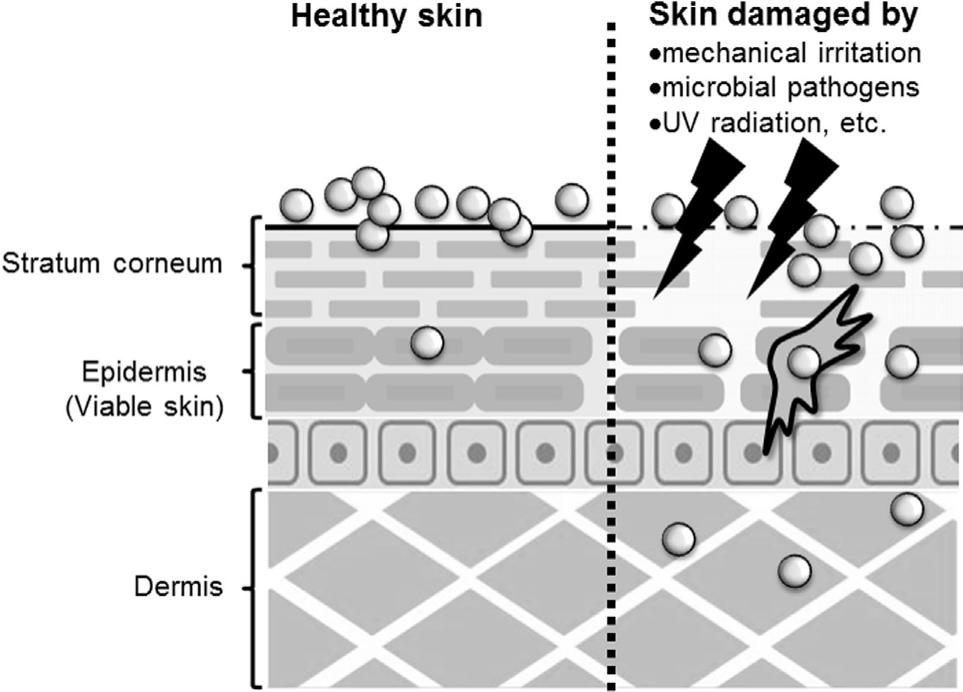
**Penetration of skin by nanomaterials**. After topical application to healthy skin, nanomaterials may penetrate to the stratum corneum or epidermis. However, after application to allergic or damaged skin, nanomaterials may penetrate to the epidermis and dermis.

Whether nanomaterials can penetrate the skin barrier *in vivo* remains controversial, although there have been several reports assessing the skin penetration of nanomaterials after topical application using both *in vitro* and *in vivo* models (Figure 1). Because TiO_2_ and ZnO nanoparticles are essential components in sunscreens, many studies have examined the penetration rate of these nanoparticles, although it should be noted that these nanoparticles are typically present in sunscreens as 30- to 150-nm aggregates. Cross et al. and Larese et al. have shown that ZnO nanoparticles with diameters of 15–40 nm, and Ag nanoparticles with a diameter of 25 nm, can penetrate the upper layers of the stratum corneum but cannot reach the deeper layers of the viable epidermis and dermis by using an *in vitro* model of human skin (33, 34). Lin et al. also showed that ZnO nanoparticles with a diameter of 10–50 nm could not penetrate healthy skin or tape-stripped skin of human volunteers (35). These reports suggest that the stratum corneum and tight junctions of skin provide an effective barrier to prevent nanomaterial penetration of healthy skin. In contrast, Gulson et al. have shown that small amounts of ZnO nanoparticles with a diameter of 19 nm can penetrate the skin after repeated application to healthy humans (36, 37). These results suggest that nanomaterials may penetrate the skin after repeated application to even healthy skin (Figure 1). However, these different results might be due to differences in the analytical methods used, in the detection sensitivity of the analytical methods, in the sample volume or skin model used, or in the type of nanomaterials and their aggregation state. For example, although Gulson et al. used ZnO particles containing the stable isotope ^68^Zn and were able to detect the concentration of ^68^Zn in the body at high sensitivity (36, 37), other groups used conventional nanomaterials and transmission electron microscopy and inductively coupled plasma mass spectrometry techniques in their studies (33–35). In addition, although transmission electron microscopy and mass spectrometry techniques are useful for evaluating the penetration of skin by nanomaterials, transmission electron microscopy is a qualitative method that cannot be used to determine the amount of nanomaterials in the skin, and inductively coupled plasma mass spectrometry is a quantitative method that cannot distinguish between nanomaterials and their dissociated ions. Therefore, the development of more sensitive qualitative and quantitative analysis methods is an important step in elucidating the penetration of nanomaterials through the skin.

The skin barrier is not always intact because skin is under constant assault every day by mechanical irritation, mechanical damage (cuts or scrapes), UV exposure, microbial pathogens, and the use of harsh soaps or cosmetic products that may contain chemical irritants (25). In addition, people with healthy skin and those with impaired skin barrier function apply sunscreens containing nanomaterials. In this regard, some studies have examined whether nanomaterials can penetrate deeply into allergic or damaged skin because nanomaterials may be able to penetrate skin with impaired barrier function. Ilves et al. showed that ZnO nanoparticles with a diameter of 20 nm could be observed in the epidermis and to a lesser extent also in the dermis of allergic skin of mice after topical application, but ZnO particles with a diameter of 240 nm were not detected (38). Similar to their observations, other studies using human skin explants with partially disrupted stratum corneum have shown that 40-nm polystyrene nanoparticles, but not 750- or 1500, and 40-nm SiO_2_ nanoparticles can translocate to the viable epidermis (39, 40). In addition, Mortensen et al. showed that quantum dot nanoparticles with a diameter of 45 nm could penetrate deep into the epidermis and dermis in sub-erythemal dose UV radiation-exposed mice (41). These reports suggest that nanomaterials generally can penetrate the deep layers of the skin, such as the epidermis and the dermis of allergic or damaged skin (Figure 1). Although the precise number of penetrated nanoparticles needs to be quantified, these findings emphasize the importance of investigating the immunomodulatory effects of nanomaterials after topical application.

Recently, hair follicles have been considered an excellent target route for drug delivery *via* skin (42). Many researchers have tried to deliver drug compounds *via* hair follicles by using particles such as liposomes (42, 43). Hair follicles have the potential to be efficient, long-term reservoirs suited for accumulation of nanomaterials. Therefore, the hair follicular pathway may be one of the penetration pathways of nanomaterials, although it remains largely unknown whether nanomaterials can indeed penetrate the skin *via* hair follicles (44, 45).

## Sensitization Potential of Nanomaterials on Skin

Allergic contact dermatitis induced by chemicals is the most frequent manifestation of skin sensitization in humans (46). It is estimated that about 4,000 chemicals have the potential to be skin sensitizers (47). Because sensitization to chemicals is sometimes induced at relatively low levels of exposure to that substance *via* skin exposure, the sensitization potential of nanomaterials might be an important potential nanotoxicity.

Park et al. showed that neither amine-modified polystyrene nanoparticles with a dimeter of 50 nm nor TiO_2_ nanoparticles (primary size <25 nm) induced skin sensitization after topical skin treatment, as assessed using a local lymph node assay (LLNA), which is a useful method for evaluating the sensitization potential of chemicals (48). Lee et al. evaluated the sensitization potential of two types of SiO_2_ nanoparticles, mesoporous SiO_2_, and colloidal SiO_2_, with diameters of about 100 nm (49). Changes in ear skin thickness after painting the skin with each nanoparticle for three consecutive days were small. In addition, these authors also found that neither nanoparticle induced skin sensitization, as assessed using a LLNA. These results suggest that the sensitization potential of many nanomaterials after topical application to healthy skin might be low. Skin painting is a typical method used to analyze the sensitization potential of chemical compounds, but nanomaterials do not easily penetrate healthy skin. Therefore, subcutaneous or intradermal administration might be useful as alternative routes for examining the sensitization potential of nanomaterials, assuming that the particles are able to penetrate allergic or damaged skin.

Epidemiological studies have suggested that sensitizer metals contained in airborne particulates may also contribute to the onset of metal allergy (50–52). Since metal nanoparticles can release metal ions, we must pay attention to the sensitization potential of not only nanoparticles but also metal ions released from metal nanoparticles. Metal allergy, which is a major cause of allergic contact dermatitis, is prevalent in the general population, and up to 17% of women are reported to suffer from it (53, 54). Nickel is the most frequent cause of metal allergy, but gold, palladium, cobalt, mercury, beryllium, chromium, and silver also have sensitization potential (54). It is suggested that metal ions from jewelry and clothes (buttons, zippers, and belt buckles) cause metal allergy *via* the activation of innate and adaptive immunity. Although it is believed that metal allergy is caused by metal-ion-induced T cells, which are generally reactive to metal ions in the major histocompatibility complex, many attempts to sensitize mice by means of simple metal-ion treatment have failed (55, 56). Moreover, while some reports have shown that metal allergy in mice can be induced by concomitant application of inflammatory stimuli, such as lipopolysaccharide (LPS) (57, 58), the skin reactions in these models could be induced by irritant inflammation rather than allergic responses (59). Nevertheless, these studies raised the possibility that other unknown factors may contribute to the onset of metal allergy.

**Figure 2:**
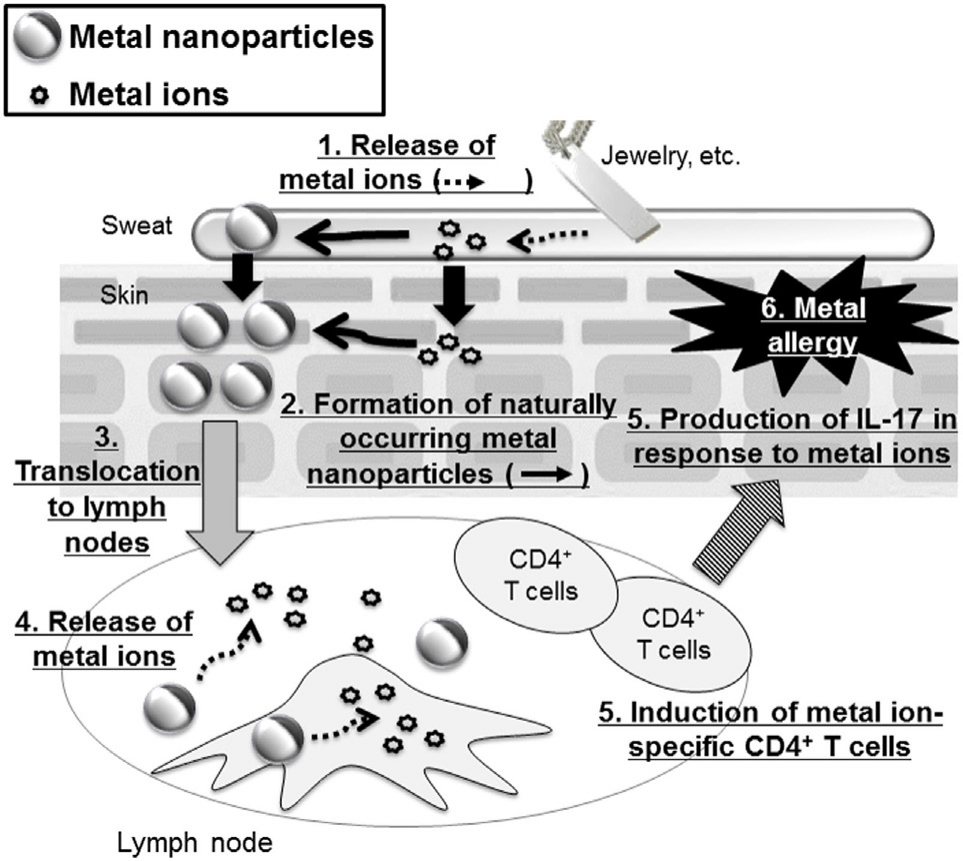
**Metal nanoparticles as a potential trigger of metal allergy**. Metal-based fashion accessories may expose the wearer to metal ions that generate metal nanoparticles. These metal nanoparticles are then translocated to the lymph nodes where they release metal ions that induce metal ion-specific CD4^+^ T cells and IL-17-mediated immune responses.

Recently, it was revealed that metal nanoparticles are generated in the environment and in our bodies naturally during our daily lives (60). For example, Glover et al. showed that metal nanoparticles were generated spontaneously from manmade objects such as earrings or metal wire, suggesting that macroscale metal objects might be a potential source of naturally occurring nanoparticles in the environment (61). In addition, naturally occurring metal nanoparticles are thought to be formed from ions *via* chemical and/or photochemical reduction of released metal ions from metal objects (61, 62). Therefore, we could be extemporaneously exposed to metal ions from metal objects when we wear metal accessories and then these ions could generate naturally occurring metal nanoparticles when we are sensitized to metal. In this regard, our group examined the contribution of metal nanoparticles to the onset of metal allergy by using Ag nanoparticles or nickel (Ni) nanoparticles with several kinds of diameters (63) (Figure 2). We showed that mice sensitized with Ag nanoparticles or nickel nanoparticles plus LPS exposure, but not with metal ions, experienced allergic inflammation in response to both metal ions and metal nanoparticles in the elicitation phase. We also showed that LPS was necessary for sensitization to metal nanoparticles. However, gold and SiO_2_ nanoparticles, which are minimally ionizable, did not induce allergic inflammation, even when co-administered with LPS. In addition, smaller metal nanoparticles had stronger sensitization potential than larger ones. We observed that CD4^+^ T cells were required for immune responses induced by metal nanoparticles and IL-17A-mediated inflammation was responsible for the allergic responses. On the basis of this study, we suggested that metal nanoparticles might play a role as a carrier, conveying metal ions to the lymph nodes for metal sensitization, because we found that smaller metal nanoparticles were transferred to the draining lymph nodes more readily than larger metal nanoparticles and metal ions. This study identifies metal nanoparticles as a new potential trigger of metal allergy and highlights the need to pay close attention to the indirect sensitization potential of metal nanoparticles when evaluating their safety.

## Combined Exposure to Nanomaterials and Other Substances

Our skin is often exposed to nanomaterials simultaneously with other chemical compounds and allergens, such as foods and pollen. Therefore, it is important to examine the possibility that skin exposure to nanomaterials contributes to allergen-induced onset of allergy.

Allergic contact dermatitis is generally induced by a chemical sensitizer. Hussain et al. showed the effect of TiO_2_ nanoparticles with a diameter of 22 nm on the sensitization potential of dinitrochlorobenzene (DNCB), a well-known skin sensitizer (64). They showed that subcutaneous injection of TiO_2_ nanoparticles before DNCB treatment increased DNCB-mediated lymph node proliferation in an LLNA and enhanced Th2-type cytokine production, whereas TiO_2_ nanoparticles alone showed no dermal sensitization. This study suggests that some nanomaterials can enhance the sensitization potential of chemical sensitizers when they penetrate the skin. Moreover, several reports have shown that topical application of nanomaterials also has an effect on skin sensitization caused by chemicals. Lee et al. showed that 3 days of consecutive skin painting with mesoporous SiO_2_ nanoparticles with a diameter of about 100 nm and 2,4-dinitroflourobenzene (DNFB) exacerbated DNFB-induced ear skin thickness and lymphocyte proliferation (49). Smulders et al. compared the effect of different topically applied nanoparticles (TiO_2_, Ag, and SiO_2_ nanoparticles) on DNCB-induced dermal sensitization by an LLNA (65). They showed that only TiO_2_ nanoparticles enhanced sensitization to DNCB by augmenting a Th2 response. Together, these reports demonstrate that some nanomaterials can enhance the potential of chemical sensitizers after either topical application or intradermal/subcutaneous injection, although the physiochemical properties of the nanomaterials (e.g., size, shape, composition, charge, and surface energy) might influence the effects. Further studies are needed to reveal the mechanisms behind these nanomaterial effects and to identify the threshold amounts that are hazardous.

It is estimated that 15–30% of children and 2–10% of adults suffer from atopic dermatitis (66). Atopic dermatitis is believed to progress to allergic rhinitis and asthma over time, which is referred to as the atopic march. Of note, the incidence of atopic dermatitis has increased gradually in industrialized countries (67, 68). Some reports have shown that co-exposure to nanomaterials and protein allergens affect atopic allergy. Yanagisawa et al. showed that intradermal injection of TiO_2_ nanoparticles of different sizes (15, 50, or 100 nm) together with mite allergen, which is a major cause of atopic dermatitis, enhanced atopic dermatitis-like skin lesions and Th2-type cytokine production, as well as total IgE and histamine levels in serum (69). They observed that the size of the TiO_2_ nanoparticles did not influence these effects. They also observed similar effects in NC/Nga mice treated with polystyrene nanoparticles (70). In this case, enhancement of allergic responses was totally depended on the size of particle, that is, the smaller polystyrene nanoparticles induced greater symptoms. We also investigated the co-exposure effects of SiO_2_ particles of different sizes (30, 70, 100, 300, or 1000 nm) and mite antigen on atopic dermatitis in NC/Nga mice (71), and found that intradermal exposure of SiO_2_ particles and mite antigen aggravated atopic dermatitis. This effect was correlated with excessive induction of total IgE and stronger systemic Th2 responses. Of note, the aggravating effects were more pronounced in the smaller SiO_2_ nanoparticle-injected mice than in the mice exposed to the larger particles.

Other reports have shown the effects of nanomaterials on atopic dermatitis after topical skin painting. Ilves et al. used a mouse model of atopic dermatitis and showed that ZnO nanoparticles with a diameter of 20 nm cause an increase in IgE production after repeated topical application. However, these nanoparticles decreased local skin inflammatory responses, such as cytokine induction, in this mouse model (38). Our group showed that topical skin painting with a mixture of SiO_2_ nanoparticles and mite allergen suppressed allergen-specific IgG production without any changes in the IgE and Th1/Th2 immune responses (72). In addition, the suppression of IgG caused severe IgE-mediated hypersensitivity in an anaphylaxis model. Interestingly, low-level IgG production was induced when the mice were exposed to allergen-SiO_2_ nanoparticle agglomerates, but not when the mice were exposed to nanoparticles applied separately from the allergen, to well-dispersed nanoparticles, or to nanoparticle agglomerates *via* routes other than the skin. Thus, agglomeration of the allergen and SiO_2_ nanoparticles may have created a “depot” effect that could control the concentration of the exposed allergen and prolong allergen exposure. Thereby, we suggest that allergen-SiO_2_ nanoparticle agglomerates facilitated IgE-biased allergic sensitization.

These reports suggest that any nanomaterials could control the immune responses induced by a chemical sensitizer or allergen on the skin of humans. However, the mechanism responsible for these effects remains unclear. In the study described above, Smulders et al. observed that titanium levels were increased in lymph node cells after topical application of TiO_2_ nanoparticles, indicating that TiO_2_ nanoparticles penetrated the skin and translocated to the lymph nodes (65). We know that nanomaterials (<100 nm) can move to the draining lymph nodes *via* lymphatic vessels, but larger particles become trapped in the tissue and tend to depot near the site of injection (73). Winter et al. showed that TiO_2_ and SiO_2_ nanoparticles induce the activation of murine dendritic cells *in vitro* by upregulating co-stimulatory molecules (74). Therefore, one of the immunomodulating mechanisms of nanomaterials might be that they move to the draining lymph nodes after topical application and activate dendritic cells in the lymph nodes. However, little is understood about how nanomaterials affect the function of immune cells such as LCs and γδ T cells in the skin. In addition, as mentioned earlier, hair follicles have recently been revealed to have important functions in regulating the trafficking of LCs and skin-resident memory T cells (29, 75), and nanomaterials are prone to accumulate in hair follicles. Future studies should include detailed investigations into the relationship between the qualitative and quantitative distribution of nanomaterials in the skin and the effects of nanomaterials on skin immune cells, keratinocytes, and hair follicles.

We also must pay attention to the interaction of nanomaterials with antigens. Nanomaterials can bind more antigen per mass unit than larger particles, because nanomaterials have a larger per unit surface area per mass than larger particles. This leads to an enhancement of antigen persistence and prolonged release, an effect referred to as the “depot effect.” It has been suggested that smaller TiO_2_ nanoparticles bind more protein antigen per mass unit than larger ones and that the depot effect on the antigen due to this binding may lead to increased antigenicity (76). Furthermore, as described below, one of the immune-activating mechanisms of aluminum salts, which are a well-known vaccine adjuvant, is believed to involve the depot effect. Therefore, the depot effect might also be one of the immune-enhancing mechanisms of nanomaterials, particularly when co-exposed with antigens.

As noted earlier, our study showed that the depot effect by allergen–SiO_2_ nanoparticle agglomerates might modulate the exposure level of allergens on the skin and could change the immune responses, even though these allergen–SiO_2_ nanoparticle agglomerates could not penetrate the skin (72). In fact, our group found that differences in the cutaneous exposure level of allergens modulates the level of allergen-specific IgG and affects susceptibility to the IgE-mediated allergic response observed in other report (77). Therefore, allergen–nanomaterial aggregates and agglomerates might modulate immune responses *via* persistent release of allergen, even though the complexes are not able to penetrate the stratum corneum or tight junctions of the epidermis. These results suggest that we must examine several types of depot effects of complexes between nanomaterials and allergens.

## The Protein Corona, Nanomaterials, and Complement

As described above, it is important to pay attention to the binding of compounds with nanomaterials. It is generally understood that nanomaterials could interact with proteins and other biomolecules contained in a biological fluid, when nanomaterials enter a biological fluid such as blood. For example, proteins bind to nanomaterials to form a coating around the surface known as the protein corona; when nanomaterials are mixed with plasma, the protein corona forms rapidly (within 30 seconds) (78). The protein composition of the corona does not seem to change markedly over time, although the concentration of a specific protein in the corona may change (78). Therefore, we must consider the possibility that the protein corona is involved in one or more of the mechanisms underlying the immunomodulating effects of nanomaterials.

The formation of the protein corona is an important factor that determines the interactions of nanomaterials with cells. Lesniak et al. reported that the protein corona surrounding nanomaterials inhibits the adhesion of nanomaterials to the cell membrane, resulting in a low internalization efficiency (79). In addition, they showed that the protein corona modulates not only the amount of nanomaterial taken up into cells but also the intracellular localization of nanomaterials within cells. Furthermore, detailed examination of the proteins within the corona has suggested that not all proteins in the protein corona modulate the cellular uptake of nanomaterials. For example, Deng et al. showed in *in vitro* studies that negatively charged gold nanoparticles bind to fibrinogen (80), and that the interaction of gold nanoparticles with fibrinogen induces fibrinogen unfolding, which promotes an interaction with the integrin receptor, Mac-1, which is expressed on macrophages. The binding and activation of Mac-1 induces inflammatory responses in macrophages. Therefore, the protein corona might contribute to the immunomodulatory effects of some nanomaterials. Indeed, if the protein corona of nanoparticles contains complement and coagulation factors, it can induce complement activation and blood clotting followed by unwanted inflammatory responses (81–84). The complement system not only works as an innate immune sensor but also plays an essential role as a trigger for inducing adaptive immunity. Although few studies have examined whether the protein corona containing complement proteins could contribute to the immunomodulatory effects of nanomaterials, some studies have suggested strategies involving the use of complement activation by nanomaterials as an adjuvant for vaccines (85, 86). Reddy et al. designed pluronic-stabilized polypropylene sulfide nanoparticles with a diameter of 25 nm that could strongly activate complement (85). They showed that the nanoparticle-conjugated antigen could induce antigen-specific immune responses. In the future, the effects of complement activation by nanomaterials on the onset of allergic responses should be investigated. In addition, few studies have systemically examined the relationship between the activation of complement and the surface properties of particles, because protein binding, including complement binding to nanomaterials, is known to depend on the physicality of nanomaterials, such as their size and surface properties (87). Elucidation of the fundamental rules that govern complement recognition of nanomaterials could help us to better predict the immunomodulatory effects of nanomaterials in the future.

## Immunomodulating Mechanisms of the Adjuvant Effects of Particles

As described above, many nanomaterials have been reported to have the potential to enhance adaptive immunity, that is, they have adjuvant effects. However, the molecular mechanisms of the adjuvanticity of nanomaterials remain largely unknown. Many particles besides nanomaterials have been reported to have adjuvant effects, such as hemozoin, which is a heme metabolite during malaria infection, chitin particles from fungal cell walls, and monosodium urate crystals released from damaged cells (88–90). Aluminum salt is a well-known particle adjuvant that is widely used throughout the world as an adjuvant for human vaccines (91). Since aluminum salts are the most studied particle with adjuvant effects, we will introduce aluminum salts as a typical example to summarize the mechanism of adjuvant effects in the context of the immunomodulatory effects of nanomaterials.

About one century ago, the usefulness of aluminum, in its potassium salt form, as a vaccine adjuvant was described for the first time (92). Since this report, several reports have shown the adjuvant effects of aluminum salts, especially aluminum oxyhydroxide, because aluminum salts induce strong antigen-specific Th2 immune responses such as the production of IL-4 and IL-5 and the induction of IgE and IgG1. Nowadays, many vaccines formulated with aluminum salts, such as the diphtheria-tetanus-pertussis vaccine, the pneumococcal conjugate vaccine, and hepatitis B vaccine, are approved by the US Food and Drug Administration (93). Recently, progress was made in revealing the mode of action of aluminum salts, although a large part of the adjuvant mechanism remains unclear.

The surface charge of aluminum salts is positive at physiological pH and aluminum salts can bind to negatively charged compounds, including protein antigens (94, 95). Therefore, the depot effect is thought to play a part in the adjuvanticity of aluminum salts. However, some studies have questioned the importance of depot effects in the adjuvanticity of aluminum salts (96–98). For example, Hutchison et al. showed that surgical removal of the injection site 2 hours after co-administration of antigen and aluminum salts had no effect on antigen-specific immune responses in mice (96). Thus, it may be that aluminum salts have additional effects that contribute to their adjuvanticity, with the depot effect being just one of the underlying mechanisms.

The NLRP3 inflammasome is gaining attention for its role in the initial stages of inflammation, such as the production of IL-1β and IL-18, which are generated in response to a number of diverse particles, including monosodium urate crystal, silica, asbestos, and aluminum salts (99–101). Some reports have shown that aluminum salts induce antigen-specific IgG1 responses that are dependent on the NLRP3 inflammasome (101, 102), although other reports suggest that the NLRP3 inflammasome is not required for the adjuvanticity of aluminum salts (103, 104). This discrepancy might stem from differences in the aluminum salts (Imject alum (101, 102) or aluminum hydroxide (103, 104)) or mice (C57BL/6 (101, 102, 104) or mixed C57BL/6–129 (103)) used in the studies, and the importance of the NLRP3 inflammasome for the adjuvanticity of aluminum salts remains controversial. Recently, it was revealed that several types of nanomaterials induce NLRP3 activation (105). For example, Simard et al. showed *in vitro* that Ag nanoparticles activate the NLRP3 inflammasome by inducing the degradation of the ER stress sensor ATF-6 (106). In addition, Sun et al. showed that NADPH oxidase-dependent NLRP3 inflammasome activation is crucial for the lung fibrosis induced by multiwalled carbon nanotubes (107). However, few studies have investigated the link between the activation of the NLRP3 inflammasome by nanomaterials and the induction of adaptive immunity. Detailed studies to test this hypothesis are expected.

Recently, Kuroda et al. explained the role of prostaglandin E2 (PGE_2_), a well-characterized proinflammatory lipid mediator, in the adjuvanticity of aluminum salts (108). Specifically, they clarified the importance of aluminum salt-induced PGE_2_ for antigen-specific IgE production, rather than IgG1 production. This information will be useful to elucidate the mechanistic basis of the aggravation effects of nanomaterials on IgE-related allergies upon co-exposure with allergens.

Recently, aluminum salt-induced cell death was reported to be an important function of adjuvanticity. Marichal et al. showed that DNA molecules released from dying host cells as a function of aluminum salt-induced innate immune responses act as damage-associated molecular patterns (DAMPs) to effectively induce adaptive immunity (109). In addition, Miki et al. showed that the interaction of apoptotic host cells, induced by aluminum salts, with CD300a, an immunoreceptor for phosphatidylserine, was important for the adjuvant effects of aluminum salts (110). Although many reports have examined the cytotoxicity of nanomaterials, few have shown the effects on the immune system of DAMPs and dead cells induced by nanomaterials. In this regard, Rabollil et al. showed that IL-1α from necrotic alveolar macrophages was important for SiO_2_ nanoparticle-induced lung inflammation (111), although the importance of this IL-1α production for the induction of adaptive immunity was not clear. Recently, Kuroda et al. also indicated that IL-1α release from alveolar macrophage death induced by aluminum salts contribute to adjuvant activity in the lungs (112). Since LPS stimulation induce alveolar macrophage death and IL-1α release (113), IL-1α release in the lungs seems an important event for immune responses in the lung. In addition, Natsuaki et al. showed that IL-1α-induced leukocyte clusters is important for efficient activation of T cells in skin (114), suggesting the essential immunological role of IL-1α in skin. Cell death might be required for allergic responses in the skin, the precise mechanisms involved in cell death in skin after exposure to nanomaterials should be investigated.

Many reports have shown the seemingly linear relationship between the biological effects of nanomaterials and their size. However, several *in vitro* studies have shown that nanomaterials with a diameter of 50 nm induce more cellular uptake or cytotoxicity compared with their larger and smaller counterparts (115, 116), suggesting the existence of size-specific nanotoxicity. Yet, few reports have shown such size-specific effects of nanomaterials *in vivo*. Therefore, further studies are needed to elucidate the size-specific immunomodulating mechanisms of nanomaterials, which might be different from those of aluminum salts.

## Future Prospects and Conclusion

It is difficult to judge whether topical application of nanomaterials to healthy skin poses a risk for disruption of immune homeostasis, because most nanomaterials cannot penetrate healthy skin. On the other hand, there is an urgent need to identify the potential of nanomaterials to cause sensitization either directly or *via* co-exposure with substances in people with allergic diseases and damaged skin. In this regard, there are many unresolved problems.

Recently, new information surfaced regarding the relationship between commensal bacteria and the host’s immune system: the commensal bacteria on the skin may in fact influence the host’s immune system (117, 118). For example, microbial diversity on skin is known to be markedly reduced in patients with atopic dermatitis, and treatment could restore this diversity (119). Therefore, we may need to consider both the direct effects of nanomaterials on the microbiota on skin and the indirect effects of nanomaterials on host immune systems *via* changes in the diversity and composition of the microbiota on skin. In addition, most bacteria have pathogen-associated molecular patterns (PAMPs), which are ligands of pattern recognition receptors, such as Toll-like receptors, Nod-like receptors, RIG-I-like receptors, and C-type lectin receptors (120). PAMPs could induce innate immunity, mediated by macrophages and dendritic cells, and activate innate immunity, such as the production of cytokines and chemokines, to induce effective adaptive immunity. Therefore, we need in-depth studies of the co-exposure effects of PAMPs and nanomaterials, in addition to the effects of DAMPs.

A range of toxicological studies have been conducted assessing various physicochemical characteristics of nanomaterials, such as particle size, surface charge, surface hydrophobicity, particle shape, and states of agglomeration and aggregation. However, the results have been inconsistent to date and definitive rules cannot yet be established. Systematic information about the relationships among the physicochemical properties and biological effects of nanomaterials is still lacking.

Recent studies have revealed that particle-induced immune responses are involved in pathological processes of chronic inflammation such as allergy. It is not too much to say that the skin is the immune sentinel of our tissues. However, the underlying mechanisms of the effects of nanomaterials are not fully understood. To establish rules governing the contributions of nanomaterials to allergic responses, we need more information, including an understanding of the molecular mechanisms of action of nanomaterials. These future studies could promote ways for us to live in harmony with nanomaterials. Furthermore, such studies would provide useful information to improve the safety and efficacy of nanomaterials used in skincare.

## Author Contributions

YY, EK, TH, YT, and KI wrote the article and prepared the figures.

## Conflict of Interest Statement

YY is employed by The Research Foundation for Microbial Diseases of Osaka University. The other authors declare that the research was conducted in the absence of any commercial or financial relationships that could be construed as a potential conflict of interest.
